# Label-Free Evaluation of Chromatin Condensation in Human Normal Morphology Sperm Using Raman Spectroscopy

**DOI:** 10.1007/s43032-021-00494-6

**Published:** 2021-04-20

**Authors:** M. Y. Jahmani, M. E. Hammadeh, M. A. Al Smadi, Marko K. Baller

**Affiliations:** 1 Department of Obstetrics and Gynecology, Assisted Reproductive Laboratory, Saarland University Hospital, Homburg, Germany; 2 Department of Informatics and Microsystems Technology, University of Applied Sciences Kaiserslautern, Campus, Zweibrücken, Germany

**Keywords:** Raman spectroscopy, Chromatin condensation, Chromomycin A3, Sperm

## Abstract

Chromatin condensation is one of the main factors essential for sperm function. Evaluation of chromatin condensation by current methods render the assessed sperm unsuitable for assisted reproduction. We examined the Raman spectra of normal morphology sperm to determine whether a non-invasive confocal Raman spectroscopy can detect spectral differences between groups having different levels of chromatin condensation. Semen samples from 85 donors who underwent ICSI were obtained. Chromomycin A3, aniline blue and acridine orange staining were performed to evaluate the protamine deficiency, histone retention and DNA fragmentation respectively. Raman spectra were obtained from 50 normal morphology sperm for each donor. Spectral analysis was performed using home written programs in LabVIEW software and samples were grouped based on chromomycin A3 staining. Raman peaks intensities at 670 cm^-1^, 731 cm^-1^, 785 cm^-1^, 858 cm^-1^, 1062 cm^-1^, 1098 cm^-1^, 1185 cm^-1^, 1372 cm^-1^, 1424 cm^-1^, 1450 cm^-1^, 1532 cm^-1^, 1618 cm^-1^ and 1673 cm^-1^ were significantly correlated with at least one of the sperm staining methods. The median intensity of the Raman peaks at 670 cm^-1^, 731 cm^-1^, 785 cm^-1^, 1062 cm^-1^, 1098 cm^-1^, 1185 cm^-1^, 1372 cm^-1^, 1424 cm^-1^, 1450 cm^-1^, 1532 cm^-1^, 1618 cm^-1^ and 1673 cm^-1^ show a significant difference between the CMA3≤41 and CMA3>41groups. The Raman spectroscopic measurements represent a promising diagnostic tool that has the ability to label-free detect sperm with chromatin abnormalities, such as improper chromatin condensation and DNA fragmentation to a certain degree similar to that of the existing staining techniques at the individual cell level.

## Introduction

According to the World Health Organization (WHO)’s criteria, infertility is the inability of couples of a reproductive age to achieve pregnancy within one year in spite of unprotected intercourse [1–3]. Roughly, 10 to 15 percent of couples worldwide have infertility problems. Female factors account for 35-40% of the cases, and male factors for about 20-40% of the cases, while 20-30% of cases are assumed to be caused by both partners or in the remaining cases due to unexplained reasons [4, 5]. Male infertility evaluation is based on the determination of semen parameters according to the WHO standard [6, 7]. This evaluation has some limitations because it does not assess all known important sperm quality aspects. Additionally, semen analysis has low predictive capability as often seen in males with normal semen parameters still being infertile [8, 9].

Several factors lie behind that phenomenon such as chromatin condensation which is necessary for sperm function and later embryonic development. Abnormal sperm chromatin condensation is associated with natural reproductive failure such as spontaneous abortion and failure of assisted reproduction procedures [10–12]. One of the most important outcomes of abnormal chromatin packaging in the sperm is the increased susceptibility to DNA fragmentation [13]. This susceptibility is confirmed in several studies which correlate the protamines deficiency as an indicator of abnormal chromatin packaging with the presence of sperm DNA fragmentation [14–17]. Therefore, any defect in the sperm chromatin will likely have a severe effect on sperm DNA integrity and its ability to participate in the fertilization process.

However, the selection of sperm during assisted reproductive technology (ART) methods like intracytoplasmic sperm injection (ICSI), depends solely on sperm shape and its motility. But there is evidence and developing consideration that sperm of normal shape are not necessarily completely functional and may feature abnormal chromatin condensation [18–20]. Fertilization by sperm with abnormal shape or with abnormal chromatin condensation may have a negative impact on early embryonic growth or may lead to the development of the genetic disease. Thus, the development of methods that can non-invasively evaluate or predict the status of chromatin condensation in living sperm would be of significant benefit and would allow for its selection and ultimately use for the ICSI procedure. Because all classical methods such as chromomycin A3, methyl green, giemsa stain and acidic aniline blue are restricted to measuring or estimating the degree of damage in a semen sample unsuitable for further therapeutic use. They render the assessed sample cells inappropriate for ART. Moreover, none give direct information about the status of viable sperm, nor would it be able to evaluate its capacity to function properly and thus achieve pregnancy.

Raman spectroscopy is a technique based on the Raman effect which studies the inelastic scattering of light by the vibrating atomic bonds. The scattered photons can either lose some of their energy (Stokes scattering) or acquire energy (anti-Stokes scattering) [21]. Generally, this method is noninvasive and nondestructive up to medium photon energies and intensities and can be applied both in vitro and in vivo under different environmental conditions. The unique interaction between the chemical bonds in the molecules and the irradiated light provides comprehensive qualitative and quantitative data about the molecules under analysis such as DNA and proteins in term of conformation, concentration, composition and intermolecular interactions [22–24]. The combination of Raman spectroscopy with a confocal microscope and its high spatial resolution allows to obtain spectra from cell substructures.

Few studies have been done on sperm using Raman spectroscopy. Interestingly, the first living cell that has been studied using Raman spectroscopy was salmon sperm. They assessed the extracted sperm DNA and found that it had a B-type conformation [25]. Huser *et al*. [26] studied the differences in the Raman spectra of sperm chromatin correlated with cell shape. They provided Raman spectroscopic evidence that DNA packaging in human sperm cells with normal shape, differs from sperm with an abnormal shape. Meister *et al*. [27] used confocal Raman microspectroscopy to evaluate the mitochondrial status of human sperm, and concentrated on the impact of ultraviolet radiation on different organelles of the sperm. They found various chemical changes in the sperm’s sub-cellular structures related to the ultraviolet light exposure time. Mallidis *et al*. [28] used Raman microspectroscopy to visualize the DNA damage in UV light treated and untreated human sperm samples. They found clear differences in the spectra obtained from the two studied groups. Sanchez *et al*. [29] employed Raman microspectroscopy to detect the oxidative DNA damage in human sperm by analyzing Raman spectra obtained from sperm samples treated with different levels of oxidative DNA damage.

In the present study we examined the Raman spectra that were obtained from normal morphology sperm in order to determine whether confocal Raman spectroscopy can detect spectral differences between the studied groups having different levels of chromatin condensation.

## Materials and Methods

### Semen samples

Semen samples were collected from donors who underwent ICSI for infertility treatment at the Prince Rashid Ben Al-Hasan Military Hospital, Irbid, Jordan. Patients gave written consent after they were approved to use these samples for research purposes based on the approval of the Royal Medical Services Human Research Ethics Committee number (8/2018). Samples were provided by masturbation after 3-6 days of sexual abstinence and processed immediately after complete liquefaction at 37°C for 30 min. Semen parameters were tested according to WHO guidelines and are summarized in Table 1.

**Table 1:** Sperm parameters statistical data

Parameters	Mean ± SD	Median	Minimum	Maximum
Chromomycin A3 (positive %)	44.95 ± 21.38	39	13	100
Aniline Blue (positive %)	37.24 ± 13.26	38	11	82
Acridine Orange (positive %)	35.88 ± 15.51	33	9	90
Age	34.34 ± 7.21	34	22	66
Volume	3.14 ± 1.5	3	0.8	7
Concentration (1x10^6^ /ml)	36.56 ± 26.25	32	0.6	150
Total Motility (motile %)	59.85 ± 20.8	65	2	90
Morphology (normal %)	8.71 ± 7.67	6	1	33

Fractions of the fresh ejaculate samples were frozen at -18°C in Jordan, transferred under cooled conditions to Germany and stored again at -18°C within 10h. This study was carried out in the Laboratory of Biochemistry and Molecular Biology of Reproductive Medicine, Department of Obstetrics and Gynecology at the University Hospital of Saarland, Homburg, Germany, and at the Department of Informatics and Microsystems Technology, University of Applied Sciences Kaiserslautern, Campus Zweibrücken, Germany.

### Sperm Purification for Raman Spectroscopy

A fraction from the raw semen samples was washed and centrifuged at 250g for 10 min in two steps: first samples were washed twice with phosphate-buffered saline (PBS). Then, the obtained sperm pellet was washed twice with deionized water. Finally, the pellet was resuspended in deionized water and stored at -20 °C until the use in the Raman measurement.

### Assessment of Chromatin Condensation by Chromomycin A3

As described by Bianchi *et al.* [30] chromatin condensation was evaluated by chromomycin A3 (CMA3) staining. A fraction of the raw semen samples was washed with Dulbecco’s Ca^2+^ -Mg^2+^ free PBS (1 volume semen: 2 volumes PBS) followed by centrifugation at 250g for 10 min. Then, the washed sperm were smeared and fixed using methanol/acetic acid, 3:1 (Carnoy’s solution) at 4°C for 5 min. Then, each slide was stained in dark for 20 min with 100 μl of CMA3 stain solution. (CMA3 stain prepared in McIlvaine buffer (pH=7.0) supplemented with 10 mM MgCl_2_ to a final concentration of 250 μg/ml). Then, each slide was rinsed in PBS buffer, dried and mounted with buffered glycerol (1:1). The evaluation of chromatin condensation each slide was examined using a fluorescence microscope (Olympus) at 100X oil immersion magnification. 500 normal morphology sperm were evaluated for each donor, by differentiating between the CMA3 positive sperm (bright yellow-stained) and CMA3 negative sperm (dull yellow-stained). The percentages of CMA3 positivity were calculated by dividing the number of sperm with positive CMA3 (protamine deficient sperm) by the total number of the evaluated sperm.

### Assessment of Chromatin Maturity (Histones Retention) by Aniline Blue

As described by Hammadeh *et al.* [31] Chromatin maturity was evaluated by aniline blue staining. A fraction of the raw semen samples was washed with PBS followed by centrifugation at 250g for 10 min. Then, the washed sperm were smeared and fixed for 30 min by 3% buffered glutaraldehyde in 0.2 M phosphate buffer (pH=7.2). Smears staining was performed by immersing the fixed slides for 5 min in 5% acidic aniline blue stain (pH=3.5). Chromatin maturity was evaluated by a light compound microscope using 100X oil immersion magnification. 200 normal morphology sperm were evaluated for each donor by distinguishing the unstained sperm (sperm with mature chromatin) from the completely or partially blue-stained sperm (sperm with retained histones). The percentages of immature sperm were calculated dividing the number of stained sperm (sperm with retained histones) by the total number of the evaluated sperm.

### Assessment of DNA Fragmentation by Acridine Orange

As described by Tejada *et al.* [32] DNA fragmentation was evaluated by acridine orange staining. A fraction of the raw semen samples was washed with PBS followed by centrifugation at 250g for 10 min. Then, the washed sperm were smeared and fixed using methanol/acetic acid, 3:1 (freshly prepared Carnoy’s solution) for overnight at room temperature. Then, the fixated smears were allowed to air dry for a few minutes. Then, each slide was stained for 5 min by adding 2-3 ml of freshly prepared acridine orange working solution 0.19 mg/ml (10 ml of acridine orange stock solution 0.1%: 40 ml of 0.1 M citric acid: 2.5 ml of 0.3 M Na_2_HPO_4_·7H_2_O: pH=2.5). Then, slides were gently washed with deionized water and covered before drying. The evaluation of chromatin DNA integrity was performed using fluorescence microscope (Olympus) at 100X oil immersion magnification. 200 normal morphology sperm were evaluated for each donor by distinguishing the green-stained sperm (sperm with intact double-stranded DNA), from yellow- or orange- or red-stained sperm (sperm with single-stranded DNA). The percentages of the DNA fragmentation for each donor were obtained by dividing the number of yellow- or orange- or red-stained sperm (sperm with single-stranded DNA) by the total number of the evaluated sperm.

### Sample Classification

Samples were classified based on the result of sperm chromatin condensation evaluation by Chromomycin A3 (CMA3) [33, 34]. Using this criterion, samples were classified into two groups based on a cut-off value determined by a ROC curve by applying the effect of CMA3 result on the fertilization rate (fertilization rate data not shown in this article).

### Spectra Acquisition

Aliquots of 20 μl of the pre-prepared sperm suspensions were smeared onto stainless steel slides and allowed to air dry. The Raman spectra of sperm were measured using a confocal Raman spectrometer (LabRAM HR, HORIBA Jobin Yvon S.A.S.), equipped with an Olympus BX41 microscope, 660 nm diode laser (100 mW), motorized notch filter selector, adjustable confocal pinhole, two switchable gratings, and CCD detector. The acquisition of all spectra were performed by 8 accumulations of 5 seconds each with slit pinhole apertures of 250 μm at 600 grooves/mm diffraction grating using the Olympus X100 objective and a wavenumber range from 600 to 1850 cm^-1^ (LabSpec 6 Software). No further processing of the spectra beyond accumulation was performed on the Instrument’s software. Spectra also were saved as comma separated values text files for further processing. A total of 50 normal morphology sperm per sample were chosen from different microscopic fields of view. To acquire each spectrum, the cell was centered and the laser directed at the postacrosomal region of the sperm head.

### Post-Acquisition Analysis

The acquired raw Raman spectra (n=4250) were automatically batch processed using home written custom programs in LabVIEW software (National Instruments LabVIEW 2019).

### Baseline Correction and Spectra Normalization

A baseline correction of the original spectra was performed to remove the spectral background caused by autofluorescence of cell components. All acquired Raman spectra were filtered by applying a 5 points baseline model using home-written program in LabVIEW software. This program automatically subtracts the background from the raw spectra without providing a significant distortion of the Raman peaks of the measured samples resulting in virtually background-free Raman spectra. Then, another program normalizes the spectra by setting the lowest intensity of the spectra to zero followed by dividing the whole spectrum by the average intensity of the whole spectrum. Then, multiplying the resulting spectra by 3 to set the maximum intensity to around 1.

### Spectral Analysis

The normalized spectra files were analyzed by home-written LabVIEW program. To determine the spectral differences among the studied groups and the variations within each group, this program was used to extract the average spectrum and standard deviation over wavenumber for each selected group. To determine the differences among the studied groups in each Raman peak, this program was used to extract the average and standard deviation of any selected Raman peak intensities.

### Statistical Analysis

Data analysis was performed using origin program (OriginPro 2020, OriginLab Corporation, Northampton, MA, USA). Data were expressed as mean ± SD, median and range. Data were tested for normality using the Anderson-Darling test. The relationship between chromomycin A3, aniline blue, acridine orange, Raman peak intensities and their standard deviation were analyzed using nonparametric correlation (Spearman's test). The Mann-Whitney U test was used to compare the two groups (CMA3≤41 versus CMA3>41). The results were considered statistically significant when the p-value was smaller than 0.05. The effect size for the Mann-Whitney U test, r, was calculated by dividing z by the square root of n (r = z / √n).

## Result

### Chromomycin A3, Aniline Blue and Acridine Orange

Three sperm functional parameters for all studied samples (n=85) were evaluated. These parameters include chromomycin A3 staining (protamine deficiency), aniline blue (histones retention) staining and acridine orange staining (DNA fragmentation). A notable variation in the percentages of the positively stained sperm in all sperm parameters was observed as shown in Table 1. Chromomycin A3 (non-condensed chromatin: protamine deficiency) positively stained percentage ranged from 13 to 100% (44.95% ± 21.38). In this test the chromomycin A3 stain, an intercalator, binds to sperm chromatin, specifically to unprotaminated DNA, resulting in bright yellow color (positive, non-condensed) for protamine deficient sperm. Normal sperm with fully protaminated DNA appear in dull yellow color (negative, condensed). Chromomycin A3 cannot bind to DNA in chromatin condensed sperm as it competes the same binding sites as protamines. Aniline blue (non-condensed chromatin: histones retention) percentage ranged from 11 to 82% (37.24% ± 13.26). In this test the aniline blue stain binds specifically to the amino acid lysine in the histones yielding in blue color (positive, non-condensed) in histones rich sperm. Normal sperm appear unstained (negative, condensed) due to the low histones content. Acridine orange staining (DNA fragmentation) percentages ranged from 9 to 92% (35.88% ± 15.51). In this test the acridine orange stain intercalates with double stranded DNA in normal sperm causing a green fluorescence (negative, intact DNA) while it binds to single stranded DNA to form aggregates in DNA fragmented sperm that fluoresce yellow, orange and/or red (positive, fragmented DNA).

### Correlations between the Assessed Functional Sperm Parameters

All three examined functional sperm parameters were tested for correlations in all studied samples (n=85). Chromomycin A3 staining was significantly positively correlated with acridine orange staining (r=0.449, p<0.001), but it has not been significantly correlated with aniline blue staining (r=0.151, p=0.166). Aniline blue staining was not significantly correlated with acridine orange staining (r=0.195, p=0.072).

### Correlation between Sperm Functional Parameters and Raman Peaks Intensities

Several Raman peaks show a high variation in their intensities among the studied samples. These peaks were 670 cm^-1^, 731 cm^-1^, 785 cm^-1^, 858 cm^-1^, 1062 cm^-1^, 1098 cm^-1^, 1185 cm^-1^, 1372 cm^-1^, 1424 cm^-1^, 1450 cm^-1^, 1532 cm^-1^, 1618 cm^-1^ and 1673 cm^-1^, the assignment of these Raman peaks for DNA or protein are shown in Table 2. The median intensities of these Raman peaks were tested for correlation with the sperm functional parameters. Table 2 shows the correlations between the Raman peaks intensities that featured a significant correlation with at least one of the functional sperm parameters.

**Table 2:** Correlations of the three examined sperm functional parameters (positive percentage stained Chromomycin A3 (CMA3), Acridine Orange (AO), Aniline Blue (AB)) with assigned Raman peak intensities. r: Spearman's correlation coefficient, p: significance levels (* significant p<0.05, ** highly significant p<0 .005)

Raman peak (cm^-1^)	Assignment / Reference	Classifi-cation		CMA3	AB	AO
670	G ring breathing modes of the DNA base [35]	DNA	r	-0.313	0.003	-0.086
			p	0.003**	0.976	0.433
731	A ring breathing modes of the DNA base [36]	DNA	r	-0.293	-0.125	-0.233
			p	0.006*	0.253	0.032*
785	T, C ring breathing modes of the DNA, backbone O-P-O [35]	DNA	r	-0.234	0.087	-0.066
			p	0.030*	0.422	0.545
858	Tyrosine [37]	Protein	r	0.334	0.191	0.221
			p	0.002**	0.079	0.042*
1062	C–O stretching vibration of deoxyribose [38]	DNA	r	-0.295	0.124	-0.060
			p	0.007*	0.256	0.5840
1098	PO_2_- stretching of DNA [35]	DNA	r	-0.610	-0.288	-0.308
			p	<0.001**	0.008*	0.004**
1185	A, C, G ring breathing modes of the DNA bases [37]	DNA	r	-0.250	-0.068	-0.112
			p	0.021*	0.533	0.307
1372	T, A, G ring breathing modes of the DNA bases [35]	DNA	r	-0.442	-0.264	-0.461
			p	<0.001**	0.014*	<0.001**
1424	Valine [39]	Protein	r	0.368	0.093	0.220
			p	<0.001**	0.396	0.043*
1450	Methylene deformation [40]	Protein	r	0.262	0.012	0.315
			p	0.015*	0.906	0.003**
1532	Histidine, Glutamate [39]	Protein	r	0.293	0.132	-0.024
			p	0.006*	0.226	0.822
1618	Tyrosine, Tryptophan [41]	Protein	r	0.356	0.002	0.183
			p	0.001**	0.979	0.092
1673	Amide I [42]	Protein	r	0.398	0.0104	0.212
			p	<0.001**	0.928	0.051
1050/1098	Calculated peak ratio		r	0.273	0.364	0.227
			p	0.011*	<0.001**	0.036*

**Fig. 1:**
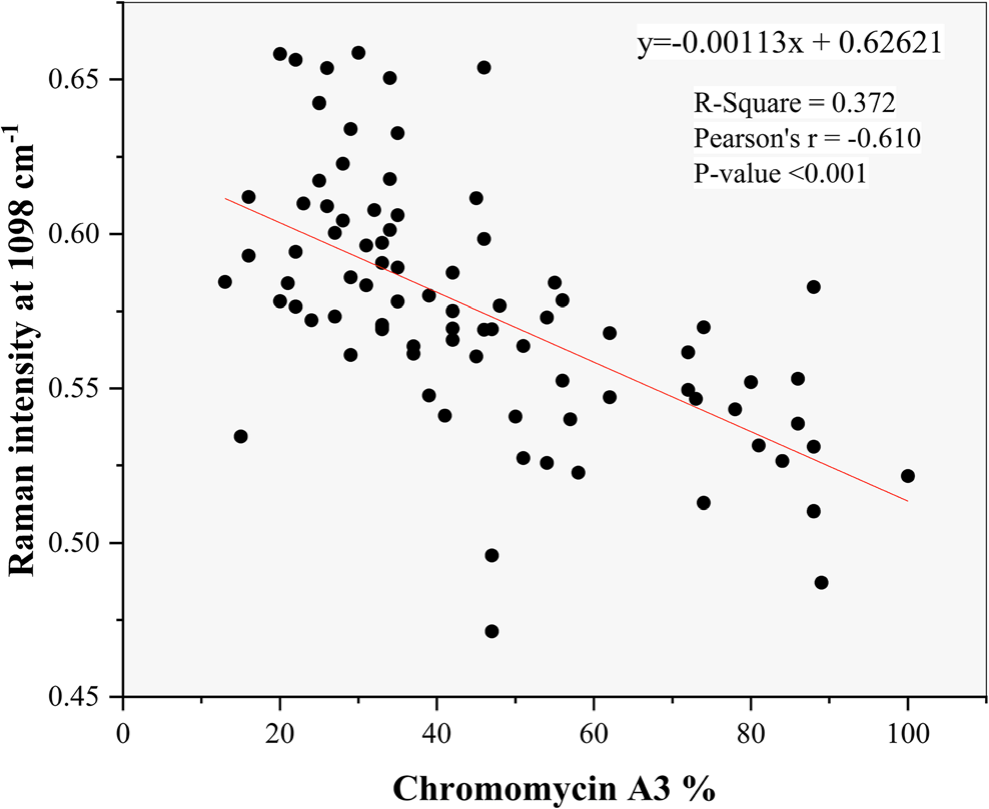
Scatter plot of the median phosphate band intensities at 1098 cm^-1^ and chromomycin A3 showing their Pearson's correlation coefficient, r^2^ and significance levels. Regression equation is given and indicated with a red line

Chromomycin A3 staining was significantly negatively correlated with the DNA related Raman peak intensities around 670 cm^-1^ (r=-0.313, p=0.003), 731 cm^-1^ (r=-0.293, p=0.006), 785 cm^-1^ (r=-0.234, p=0.030), 1062 cm^-1^ (r=-0.295, p=0.007), 1098 cm^-1^ (r=-0.610, p<0.001), 1185 cm^-1^ (r=-0.250, p=0.021) and 1372 cm^-1^ (r=-0.442, p<0.001) as shown for 1098 cm^-1^ in Fig. 1. It was significantly positively correlated with the protein related Raman peak intensities around 858 cm^-1^ (r=0.334, p=0.002), 1424 cm^-1^ (r=0.368, p<0.001), 1450 cm^-1^ (r=0.262, p=0.015), 1532 cm^-1^ (r=0.293, p=0.006), 1618 cm^-1^ (r=0.356, p=0.001) and 1673 cm^-1^ (r=0.398, p<0.001). Aniline blue staining was significantly negatively correlated with the DNA related Raman peak intensity around 1098 cm^-1^ (r=-0.288, p=0.008) and 1372 cm^-1^ (r=-0.264, p=0.014). Acridine orange staining was significantly negatively correlated with the DNA related Raman peak intensities around 731 cm^-1^ (r=-0.233, p=0.032), 1098 cm^-1^ (r=-0.308, p=0.004), 1372 cm^-1^ (r=-0.461, p<0.001) and 1424 cm^-1^ (r=-0.220, p=0.043), while it was significantly positively correlated with the protein related Raman peak intensity around 858 cm^-1^ (r=0.221, p=0.042) and 1450 cm^-1^ (r=0.315, p=0.003). Finally, the Raman peak intensities ratio (1050 cm^-1^/1098 cm^-1^) was significantly positively correlated with the percentage of the chromomycin A3 positive (r=0.273, p=0.011), aniline blue staining (r=0.364, p<0.001) and acridine orange (r=0.227, p=0.036).

### Samples Classification

Different thresholds values were tested to determine a cut-off value of CMA3 for donor group differentiation regarding fertilization rates, and 41% (positive) was used. Samples were classified into two groups: CMA3≤41% and CMA3>41%. The ROC result for this threshold yields an area under the curve of 0.8589 with 87.8% specificity and 81.8% sensitivity. This means that CMA3 is an excellent diagnostic test in predicting fertilization rates. The median fertilization rates of the CMA3≤41% and CMA3>41% differed significantly at 82 % ± 15.5 for the CMA3≤41% group vs 57 % ± 19.7 for the CMA3>41% group (Mann-Whitney, n=85, p<0.001, effect size r= 0.62)

### CMA3≤41 and CMA3>41 Spectra

**Fig. 2:**
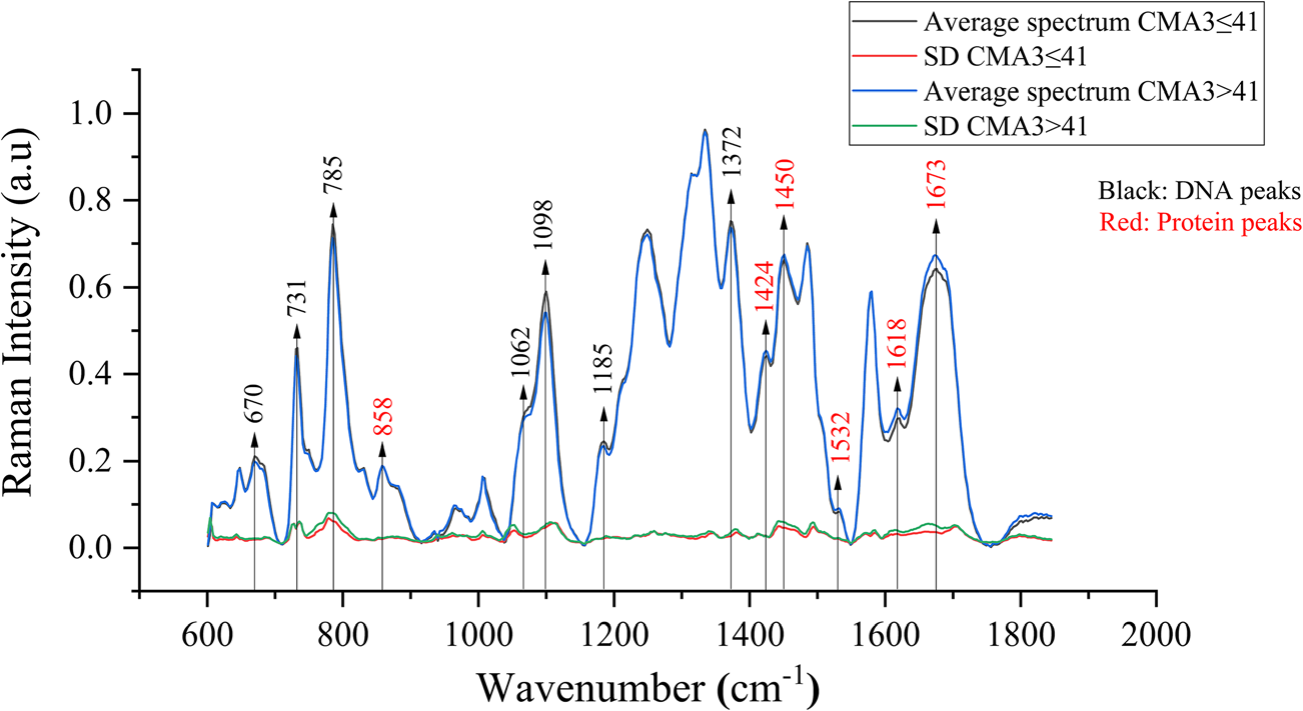
Average Raman spectra of CMA3≤41 versus CMA3>41 groups. Peaks that show a significant difference between the two groups are marked

**Fig. 3:**
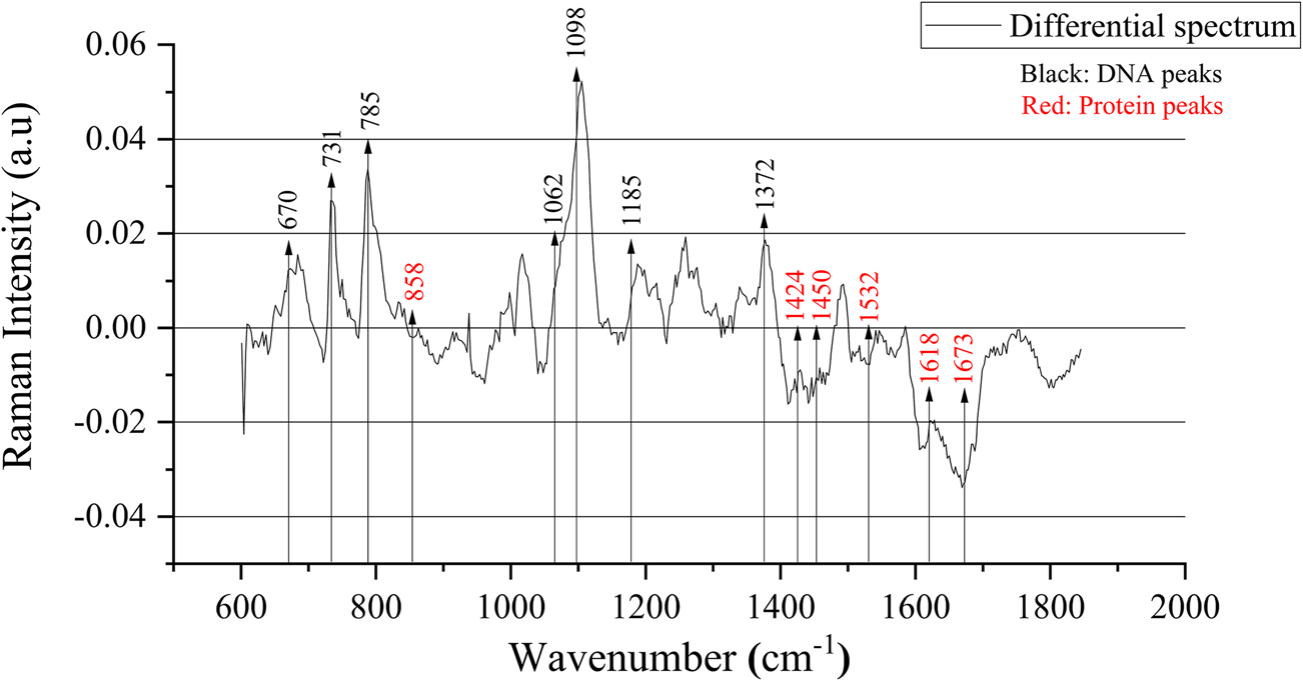
Differential spectrum for the averages of CMA3≤41 group minus CMA3>41 group. Peaks that show a significant difference between the two groups are marked

**Fig. 4:**
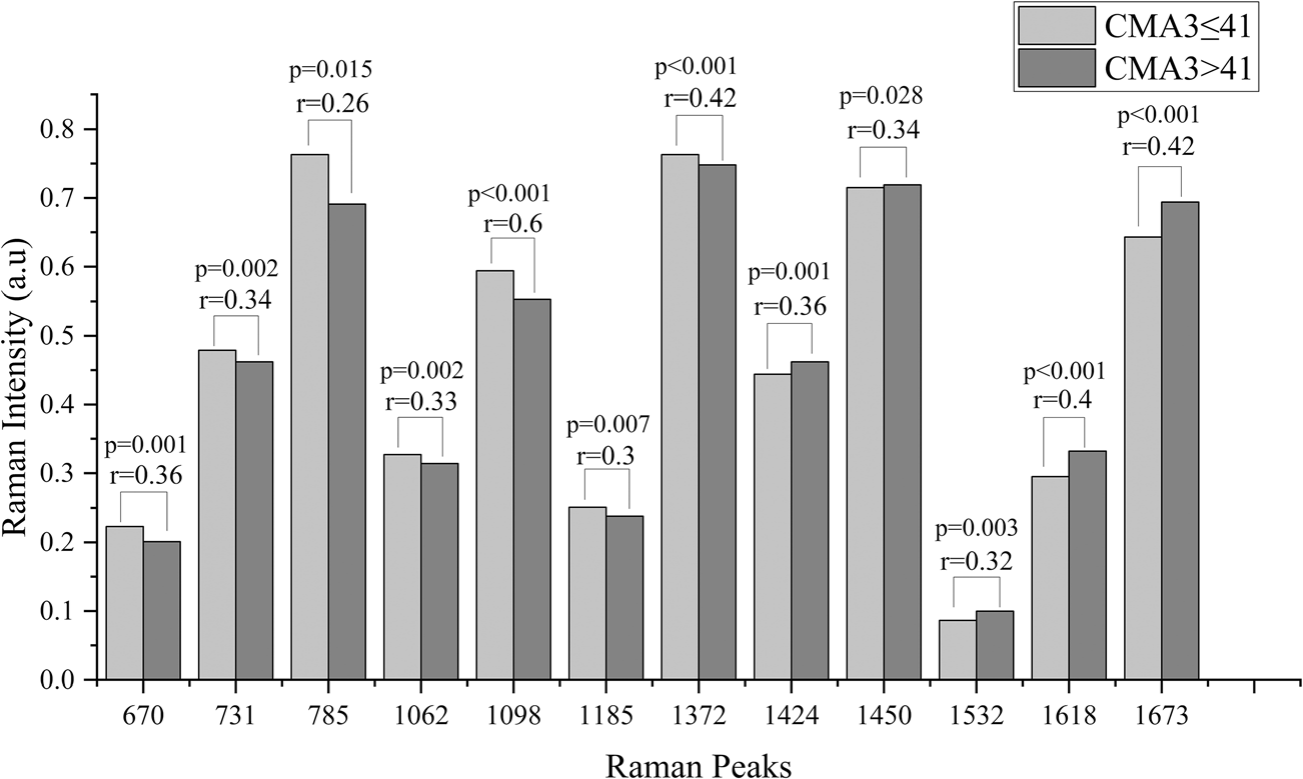
Bar plot of the median intensity of the Raman peaks that show a significant difference between the CMA3≤41 and CMA3>41 groups. The indicated r-values represent the effect size of the corresponding Raman peaks

**Fig. 5:**
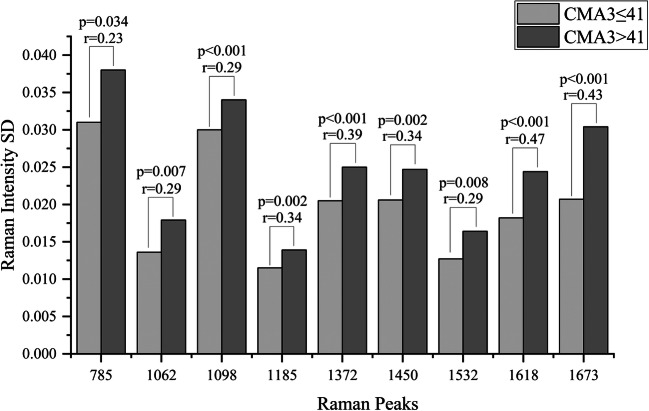
Bar plot of the median of the Raman peaks standard deviations that show a significant difference between the CMA3≤41 and CMA3>41 groups. The indicated r-values represent the effect size of the corresponding Raman peaks SD

Figure 2 shows the average Raman spectrum and the standard deviation of the CMA3≤41 and the CMA3>41 groups. The differential spectrum of the averages of CMA3≤41 group minus CMA3>41 group shows that the DNA Raman peaks are in the positive range, while the protein Raman peaks are in the negative range as illustrated in Fig. 3. 12 Raman peaks (670 cm^-1^, 731 cm^-1^, 785 cm^-1^, 1062 cm^-1^, 1098 cm^-1^, 1185 cm^-1^, 1372 cm^-1^, 1424 cm^-1^, 1450 cm^-1^, 1532 cm^-1^, 1618 cm^-1^ and 1673 cm^-1^) show a significant difference between the CMA3≤41 and CMA3>41 groups in their median peaks intensities as illustrated in Fig. 4. Raman peaks at 670 cm^-1^ (CMA3≤41 (0.223±0.013), CMA3>41 (0.201±0.015), p=0.001), 731 cm^-1^ (CMA3≤41 (0.479±0.029), CMA3>41 (0.462±0.028), p=0.002), 785 cm^-1^ (CMA3≤41 (0.763±0.05), CMA3>41 (0.691±0.064), p=0.015), 1062 cm^-1^ (CMA3≤41 (0.327±0.018), CMA3>41 (0.314±0.024), p=0.002), 1098 cm^-1^ (CMA3≤41 (0.594±0.031), CMA3>41 (0.553±0.033), p<0.001), 1185 cm^-1^ (CMA3≤41 (0.251±0.015), CMA3>41 (0.238±0.015), p=0.007) and 1372 cm^-1^ (CMA3≤41 (0.763±0.0123), CMA3>41 (0.748±0.0171), p<0.001) showed higher medians intensities in the CMA3≤41 group, while the Raman peaks at 1424 cm^-1^ (CMA3≤41 (0.444±0.019), CMA3>41 (0.462±0.017), p=0.001), 1450 cm^-1^ (CMA3≤41 (0.715±0.0137), CMA3>41 (0.719±0.0213), p=0.028), 1532 cm^-1^ (CMA3≤41 (0.0864±0.0153), CMA3>41 (0.0998±0.0139), p=0.003), 1618 cm^-1^ (CMA3≤41 (0.295±0.025), CMA3>41 (0.332±0.031), p<0.001) and 1673 cm^-1^ (CMA3≤41 (0.643±0.027), CMA3>41 (0.694±0.041), p<0.001) showed higher medians intensities in the CMA3>41 group. Generally, the CMA3>41 group shows a higher variation than the CMA3≤41 group as indicated by the higher standard deviation values throughout the spectra. This is most pronounced at the Raman peaks intensities at 785 cm^-1^, 1062 cm^-1^, 1098 cm^-1^, 1185 cm^-1^, 1372 cm^-1^, 1450 cm^-1^, 1532 cm^-1^, 1618 cm^-1^ and 1673 cm^-1^ showing significant differences in their standard deviations as illustrated in Fig. 5.

## Discussion

The evaluation of male infertility has become more important and revealing with the availability of new diagnostic and treatment methods [43]. Sperm chromatin condensation is a primary factor affecting male fertility. It is evident that the typical composition of sperm chromatin is a fundamental factor in maintaining DNA integrity [44]. Any defect in sperm chromatin will probably have a severe effect on sperm DNA integrity and its ability to participate in the fertilization process.

To establish a new diagnostic technique based on the Raman spectroscopy that is able to distinguish between sperm with normal and abnormal chromatin condensation it is essential to understand the Raman spectra of the sperm components and Raman spectral differences between normal sperm and sperm with chromatin condensation abnormalities.

Previous studies analyzed Raman spectra and correlated spectral features to various properties [25, 26, 39–42, 45–52]. The presence of the Raman peak around 670 cm^-1^ represents the ring breathing mode of guanine, indicates that the DNA backbone was in B-form conformation [53]. Raman peaks around 731 cm^-1^ and 785 cm^-1^, which are the ring breathing modes of the nitrogen bases in the DNA nucleotides, are correlated to the DNA content and can be used to differentiate between sperm with X- or Y-chromosome [40, 50].

The results of the current study showed a significant difference in the Raman peaks median intensities of 731 cm^-1^ and 785 cm^-1^ that were significantly higher in the low chromomycin A3 (CMA3≤41) group compared to the high chromomycin A3 (CMA3>41) group (731 cm^-1^ (CMA3≤41 (0.479±0.029), CMA3>41 (0.462±0.028), p=0.002), 785 cm^-1^ (CMA3≤41 (0.763±0.05), CMA3>41 (0.691±0.064), p=0.015)) with medium effect size of r=0.34 for 731 cm^-1^ and small effect size of r=0.26 for 785 cm^-1^. This indicates that chromatin condensation quality could affect the sperm’s relative DNA content. The highly condensed chromatin in the low chromomycin A3 (CMA3≤41) group produces more intense DNA Raman signals due to the presence of highly compacted DNA in the laser beam path compared to the high chromomycin A3 (CMA3>41) group in which the loosely compacted DNA produces less intense DNA Raman signals. The highly compacted chromatin protects the sperm DNA against DNA fragmentation factors such as nucleases and polymerases [54, 55] or oxidative stress [56] and this could be confirmed by the significant difference of the DNA fragmentation between the two groups (CMA3≤41 (27±10.93), CMA3>41 (42±15.74), p<0.001). It is obvious that the highly condensed chromatin in the low chromomycin A3 (CMA3≤41) group seems to be more protected compared to the high chromomycin A3 (CMA3>41) group. The symmetric stretching vibration of the phosphate (PO_4_) peak around 1098 cm^-1^ showed the same trend and confirmed the previous finding. It was significantly higher in the low chromomycin A3 (CMA3≤41) group compared to the high chromomycin A3 (CMA3>41) group (CMA3≤41 (0.594±0.031), CMA3>41 (0.553±0.033), p<0.001) with a large effect size r=0.6. It was reported that Raman peaks intensity and position are affected by nucleotide composition and sequence [57], and the Raman phosphate (PO_4_) peak around 1098 cm^-1^ is the least affect one [58]. Therefore, this peak is often used to estimate DNA content [59]. Based on these grounds and the large effect size of the phosphate peak, the DNA contents dramatically differ between the low and the high chromomycin A3 groups. Therefore, the low chromomycin A3 (CMA3≤41) group has more DNA occupied in the irradiated zone indicating that the DNA is denser and more compacted compared to the high chromomycin A3 (CMA3>41) group. Regarding the Raman peak intensity of adenine vibration mode around 731cm^-1^, it was significantly higher in the low chromomycin A3 positive group compared to the high chromomycin A3 positive group. A similar finding was reported by Hud *et al*. [60] in which they found the intensity of this Raman peak being significantly higher in the salmine- and polyarginine-DNA complexes compared to the native B-form DNA. The increased intensity was higher in the salmine-DNA complex than the polyarginine-DNA complex. This is not surprising and indicates that the packaging efficiency of protamines is higher compared to polyarginine. Also, this provides evidence that naturally occurring chromatin condensation during spermiogenesis results in more compacted chromatin compared to that produced by incorporating protamines or polyarginine to DNA in vitro [26].

The same trend was observed in the Raman peaks around 1185 cm^-1^ and 1372 cm^-1^ in which the median intensities of these Raman peaks were significantly higher in the low chromomycin A3 (CMA3≤41) group compared to the high chromomycin A3 (CMA3>41) group (1185 cm^-1^ (CMA3≤41 (0.251±0.015), CMA3>41 (0.238±0.015), p=0.007) and 1372 cm^-1^ (CMA3≤41 (0.763±0.0123), CMA3>41 (0.748±0.0171), p<0.001)) with medium effect size r=0.3 for 1185 cm^-1^ and r=0.42 for 1372 cm^-1^. Several studies identify these Raman peaks and assigned them to ring breathing modes of DNA nitrogen bases [26, 40, 41, 45–49, 51]. These results confirmed the effect of chromatin condensation quality on the sperm relative DNA content. These changes in the Raman activities of these DNA nitrogen bases could be caused by DNA damage [61].

On the other hand, Huser *et al*. [26] linked the highly intense Raman peak (which is very weak in their result) at 785 cm^-1^ with protamines packaging efficiency. With low intensity indicating highly packaged chromatin. The result of the current study showed a significant negative correlation (r=-0.234, p=0.030) between this peak and the chromomycin A3 positivity indicating that the intensity of this peak is significantly decreased in poorly condensed chromatin (785 cm^-1^ (CMA3≤41 (0.763±0.05), CMA3>41 (0.691±0.064), p=0.015)). This result is in the same line as Hud *et al*. [60] in which they found the intensity of 785 cm^-1^ Raman peak being higher in the salmine-DNA complex compared to the native B-form DNA. This peak results from the contribution of cytosine and sugar-phosphate backbone vibrational modes. The increased intensity could be explained by the disturbance of the vibrational modes that occur as a result of binding to arginine residues in the protamine [60]. Amaral *et al*. [45] found the intensity of this peak is the lowest in sea urchin compared to the other studied species. Protamines are absent in sea urchin [62].

The Raman peak of the methylene deformation mode around 1450 cm^-1^ was significantly higher in the high chromomycin A3 (CMA3>41) group compared to the low chromomycin A3 (CMA3≤41) group (CMA3≤41 (0.715±0.0137), CMA3>41 (0.719±0.0213), p=0.028) with medium effect size r=0.34. This peak is associated with the protein and lipids content in the cell [26, 29, 52]. This can be further confirmed by the absence of this peak in the Raman spectrum of purified salmon sperm DNA compared to that of the intact salmon sperm [25]. A similar finding was observed in this peak by Sanchez *et al*. [29]. They found the intensity of this Raman peak increased with oxidatively induced DNA fragmentation. This is not surprising, because DNA fragmentation is one of the main consequences of the abnormal chromatin condensation, and chromatin condensation evaluated by chromomycin A3 is highly correlated with the oxidative DNA fragmentation [63, 64]. Also, the result of the current study showed that DNA fragmentation evaluated by acridine orange was significantly positively correlated with the Raman peak intensity around 1450 cm^-1^ (r=0.315, p=0.003). Two possible explanations could be offered here. The first one is based on the lipids contribution in this peak. Unsaturated fatty acids are the main component of the biological membranes and are very vulnerable to oxidative attack. Therefore, alteration in the lipids content can be associated or may be caused by lipid peroxidation, which is a well-recognized indicator of oxidative DNA fragmentation [29, 63]. The second explanation is based on the protein contribution in this peak. As indicated in the result, sperm with low chromatin condensation quality (CMA3>41) have a higher relative protein content compared to sperm with high chromatin condensation quality (CMA3≤41). This could be explained by the presence of other nuclear proteins such as histones or transition proteins. These proteins are more loosely bound to the DNA and larger compared to protamines resulting in less condensed chromatin [26]. They are also less efficient in protecting the DNA against damaging factors. This could be partially confirmed by the result of aniline blue test, in which the retained histone was higher in CMA3>41 group compared to the CMA3≤41 group, but not significantly (CMA3≤41 (35±11.66), CMA3>41 (42±14.41), p=0.058).

The same trend was observed in the Raman peaks around 1424 cm^-1^, 1532 cm^-1^, 1618 cm^-1^ and 1673 cm^-1^ in which the median intensities of these Raman peaks were significantly higher in the high chromomycin A3 (CMA3>41) group compared to the low chromomycin A3 (CMA3≤41) group (1424 cm^-1^ (CMA3≤41 (0.444±0.019), CMA3>41 (0.462±0.017), p=0.001), 1532 cm^-1^ (CMA3≤41 (0.0864±0.0153), CMA3>41 (0.0998±0.0139), p=0.003), 1618 cm^-1^ (CMA3≤41 (0.295±0.025), CMA3>41 (0.332±0.031), p<0.001) and 1673 cm^-1^ (CMA3≤41 (0.643±0.027), CMA3>41 (0.694±0.041), p<0.001)) with medium effect sizes r=0.36 for 1424 cm^-1^, r=0.32 for 1532 cm^-1^, r=0.4 for 1618 cm^-1^ and r=0.42 for 1673 cm^-1^. Several studies identify these Raman peaks and assigned them to protein [39–42, 45, 47, 49, 51]. The elevated intensities of these protein Raman peaks confirmed the previously discussed idea about the presence of other proteins in the low quality condensed group such as histone and transition proteins that bound to the DNA more loosely than protamines.

Huser *et al*. [26] plotted two-dimensional distribution of the Raman peaks intensities ratio 785 cm^-1^/1098 cm^-1^ and 1445 cm^-1^/1098 cm^-1^ in order to discriminate between normal morphology sperm from abnormal sperm. The ratio distribution was not accurate and most abnormal morphology sperm were found in the normal morphology range. Mallidis *et al*., Huang *et al*. [28, 52] and in the current study similar plots were drawn but, no similar results were observed. As a reminder, all spectra in the current study were acquired from normal morphology sperm and it is therefore expected to not find any pattern based on the same hypothesis. Raman spectroscopy is an accurate technique, and these obvious variations in the result could be caused by the preparation method employed by Huser *et al*. [26], in which they used a chemically treated amembranous sperm, in addition to extremely low sample size (n=1).

The results of the current study showed that the Raman peak intensities ratio (1050 cm^-1^/1098 cm^-1^) was significantly positively correlated with the percentage of the chromomycin A3 positivity (r=0.273, p=0.011) and this ratio was significantly higher in high chromomycin A3 (CMA3>41) group compared to the low chromomycin A3 (CMA3≤41) group (CMA3≤41 (0.544±0.036), CMA3>41 (0.561±0.05), p=0.039). A similar trend was found for DNA fragmentation evaluated by acridine orange. DNA fragmentation was significantly correlated with Raman peak intensities ratio (1050 cm^-1^/1098 cm^-1^) (r=0.227, p=0.036). This result is in agreement with that of Sanchez *et al*. [29]. They found the Raman peak intensities ratio (1050 cm^-1^/1098 cm^-1^) being correlated with induced oxidative DNA fragmentation evaluated by flow cytometry also based on acridine orange. The same trend was observed by Mallidis *et al*. [28]. They reported that the Raman peak intensities ratio (1050 cm^-1^/1098 cm^-1^) was increased with UV-induced DNA fragmentation. As mentioned before, the phosphate (PO_4_) Raman peak around 1098 cm^-1^ is the least variable DNA peak and is assumed not to be affected by nucleotide composition or sequence [58]. As a consequence, this spectral ratio difference represents a distortion in the chemical bonds between DNA bases and consequently affecting conformation and chromatin condensation [27, 65]. The increased intensity of the C–O stretching vibration of deoxyribose peak around 1050 cm^-1^ could be due to the changes in the groups and their force-bearing environment resulting from covalent bonds breakages between the deoxyribose and phosphate groups [38]. The results of Sanchez *et al*. and Mallidis *et al*. [27, 29] have a main difference compared to the current study. Their data show a highly elevated intensity around 1050 cm^-1^ and it appears as a separated peak. In the current study and other several studies [27, 40, 45, 52] this peak is actually rather small and shows as a shoulder of the major phosphate peak as illustrated in Fig. 2. The separate peak around 1050 cm^-1^ they observed could be caused by the extensive DNA fragmentation caused by their sample preparation methods or could alternatively partially be explained by the substrate used during the spectral acquisition. They used quartz that contributes with a significant Raman signal in the vicinity of this Raman peak at 1050 cm^-1^ (own data, not shown). This spectral background contribution might not have been fully corrected by the authors.

As indicated in the results, the Raman peaks intensities show differential variability among the studied samples, as indicated in their standard deviations values. These variations were significantly higher in high chromomycin A3 (CMA3>41) group compared to the low chromomycin A3 (CMA3≤41) group. The magnitude of Raman peak intensity is directly related to the concentration of corresponding molecules of that peak. Therefore, these variations reflect variations in the biochemical content of the assessed sperm. It is known that sperm either possess a X-chromosome or a Y-chromosome that contain different amounts of DNA. Although this difference is small, it is reported that Raman spectroscopy can differentiate between X or Y bearing chromosome sperm [40, 50]. These generally observed variations could be partially explained by the type of sex chromosome. But the main difference could be resulting from the presence of subpopulations of sperm in the samples under analysis. These variations indicate that these samples contain sperm that have different chemical compositions. This is most likely caused by different maturation levels reflecting some hidden anomalies in spermatogenesis that could produce normal morphology sperm with immature chromatin.

In summary, the spectral analysis of Raman peaks indicates that the Raman DNA related peaks intensities and with it the DNA densities decreased when chromatin condensation quality decreased, while the Raman proteins related peaks intensities and with-it protein densities increased when the chromatin condensation quality decreased.

## Conclusion

The result of this study indicate that Raman spectroscopic measurements represent a promising diagnostic tool that has the ability to detect sperm with chromatin abnormalities such as improper chromatin condensation and DNA fragmentation to a certain degree similar to that of the existing staining techniques at the individual cell level. Unlike the currently used chromatin integrity tests that destroy the sample under analysis, Raman spectroscopy should have the ability to detect chromatin integrity noninvasively in living sperm. Therefore, Raman spectroscopy represents a promising technique that could be accompanied with an ICSI procedure and used to select sperm with proper chromatin condensation and intact DNA. But, this ultimate goal still needs further evaluation, e.g. establishing laser power and exposure time conditions that are actually noninvasive for the living sperm.

## Data Availability

The data underlying this article cannot be shared publicly due to patient data privacy protection required by the ethics approval. The data will be shared on reasonable request to the corresponding author.
